# Novel protein-repellent and antibacterial polymethyl methacrylate dental resin in water-aging for 6 months

**DOI:** 10.1186/s12903-022-02506-6

**Published:** 2022-10-29

**Authors:** Li Cao, Xianju Xie, Wenqi Yu, Hockin H. K. Xu, Yuxing Bai, Ke Zhang, Ning Zhang

**Affiliations:** 1 Department of Orthodontics, School of Stomatology, Capital Medical University, Beijing, 100050 China; 2 Department of Endodontics, Periodontics and Prosthodontics, University of Maryland Dental School, Baltimore, MD 21201 USA

**Keywords:** Novel polymethyl methacrylate resin, Mechanical properties, Antibacterial and protein-repellent effects, Water-aging

## Abstract

**Background:**

The present study aimed to develop a novel protein-repellent and antibacterial polymethyl methacrylate (PMMA) dental resin with 2-methacryloyloxyethyl phosphorylcholine (MPC) and quaternary ammonium dimethylaminohexadecyl methacrylate (DMAHDM), and to investigate the effects of water-aging for 6 months on the mechanical properties, protein adsorption, and antibacterial activity of the dental resin.

**Methods:**

Four groups were tested: PMMA control; PMMA + 3% MPC; PMMA + 1.5% DMAHDM; and PMMA + 3% MPC + 1.5% DMADDM in acrylic resin powder. Specimens were water-aged for 1 d, 3 months, and 6 months at 37 ℃. Their mechanical properties were then measured using a three-point flexure test. Protein adsorption was measured using a micro bicinchoninic acid (BCA) method. A human saliva microcosm model was used to inoculate bacteria on water-aged specimens and to investigate the live/dead staining, metabolic activity of biofilms, and colony-forming units (CFUs).

**Results:**

The flexural strength and elastic modulus showed a significant loss after 6 months of water-ageing for the PMMA control (mean ± SD; *n* = 10); in contrast, the new protein repellent and antibacterial PMMA resin showed no strength loss. The PMMA–MPC–DMAHDM-containing resin imparted a strong antibacterial effect by greatly reducing biofilm viability and metabolic activity. The biofilm CFU count was reduced by about two orders of magnitude (*p* < 0.05) compared with that of the PMMA resin control. The protein adsorption was 20% that of a commercial composite (*p* < 0.05). Furthermore, the PMMA–MPC–DMAHDM-containing resin exhibited a long-term antibacterial performance, with no significant difference between 1 d, 3 months and 6 months (*p* > 0.05).

**Conclusions:**

The flexural strength and elastic modulus of the PMMA–MPC–DMAHDM-containing resin were superior to those of the PMMA control after 6 months of water-ageing. The novel PMMA resin incorporating MPC and DMAHDM exhibited potent and lasting protein-repellent and antibacterial properties.

## Background

Dental plaque accumulates on the surface of teeth or restorative materials as a biofilm, which maintains microbial homeostasis [1–3]. Significant changes in the local environment could break the balanced status of the microbial community, resulting in oral diseases, such as caries [2, 4]. Dental caries is more serious in adults who wear removable dentures and children who wear removable appliances or Hawley retainers [5]. With the increase in the aging population and the rise in the incidence of malocclusion, oral dentures and orthodontic appliances have become more widely used. Thus, there is an urgent need for new dental materials that can inhibit bacteria growth and reduce virulence to decrease the incidence of oral diseases.

Resins based on polymethyl methacrylate (PMMA) are widely used in prosthodontics and orthodontics to fabricate removable dentures, occlusal splints, functional appliances, or Hawley retainers [6–8]. PMMA can exhibit sufficient esthetic, mechanical and physiochemical properties and moldability [9]. With the development of computer-aided design and computer-aided manufacturing (CAD/CAM) technology, PMMA resin shows higher mechanical resistance, better surface smoothness and better color stability [10–13]. However, the PMMA-based acrylic resin base has a complex structure, large volume, and relatively high water absorption capacity, which decreases the flow of saliva and facilitates the accumulation of dental plaque [14–16]. Cleaning technologies are not reliable; therefore, various attempts have been made to develop antibacterial acrylate resins that do not rely on patients’ compliance [17, 18]. The newly designed dental materials should have appropriate mechanical strength, sufficient protein-repellent and anti-bacterial properties, and long-lasting service time. The greater the mechanical strength of the appliance, the longer its service life. If the appliance has anti-protein adhesion and antibacterial effects, it will greatly reduce the occurrence of oral diseases.

To achieve the above goals, many additives have been incorporated into the denture base for antimicrobial modification. Compared with the soluble antibacterial agent such as Ag, F and ZnO, the polymeric materials have the advantages of non-volatile and chemically stable [19–23]. Generally, polymers include synthetic polymers and natural polymers. Materials based on natural polymers have good biocompatibility, and can be degraded in biological systems by the hydrolysis of bonds along the polymer chain followed by oxidation [24]. Among them, quaternary ammonium methacrylates (QAMs) are probably the most studied polymeric biocides [25]. In our previous work, QAMs were incorporated into composites, bonding agents, primers, and adhesives, and were shown to be effective in reducing bacterial and microbial growth [26–29]. Dimethylaminohexadecyl methacrylate (DMAHDM), a new kind of quaternary ammonium monomer, has been shown to have the strongest antibacterial activity among tested QAMs [30]. Another way to reduce the pathogenicity of dental plaque is to reduce protein adhesion. Less protein adsorption will reduce bacterial adhesion and thus reduce the formation of dental plaque. 2-methacryloyloxyethyl phosphorylcholine (MPC), which has a phospholipid polar group in its side chain, exhibits excellent protein repelling and anti-bacterial adhesion effects [23]. Recently, MPC was combined with DMAHDM to synthesize a new acrylic resin with a combination of antibacterial and protein-repellent abilities [31]. For various dental applications, a prerequisite for a new material is to survive long-term hydrolytic attacks and aqueous degradation. However, a literature search revealed no report on protein-repellent and antibacterial PMMA resin containing MPC and DMAHDM subjected to long-term water-ageing for 6 months to assess its mechanical properties. Furthermore, there has been no report on whether the protein-repellent and antibacterial functions of the PMMA resin would be lost after long-term water-aging.

Therefore, the objectives of this study were to develop a novel protein-repellent and antibacterial PMMA resin, and to investigate the effects of water-aging for 6 months on its mechanical properties, protein adhesion, and antibacterial activity. We hypothesized that: (1) Incorporating MPC and DMAHDM into a PMMA resin would not compromise its strength during 180 days of water-aging; and (2) the PMMA resin containing MPC and DMAHDM would greatly decrease protein adsorption and bacterial viability compared to the acrylate resin control, even after 180 days of water-aging.

## Materials and methods

### Incorporation of Protein-Repellent DMAHDM

The acrylic resin Nature Cryl™ MC (GC America Inc., Alsip, IL, USA) was obtained commercially. According to the manufacturer’s instructions, the acrylic resin powder and liquid were mixed at a ratio of 1:0.5. Samples were prepared following ISO 24026–2:2020 [32] (Plastics — Poly (methyl methacrylate) (PMMA) moulding and extrusion materials — Part 2: Preparation of test specimens and determination of properties) standards. The antibacterial monomer DMAHDM, with an alkyl chain length of 16, was synthesized using a modified Menschutkin reaction method [32, 33]. The advantage of this method is that the reaction product is produced quantitatively and does not require further purification [27]. Briefly, 10 mmol of 2-(dimethylamino) docecane (DMAEMA, Sigma-Aldrich, St. Louis, MO, USA), 10 mmol of 1-bromohexadecane (BHD, TCI America, Portland, OR, USA), and 3 g of ethanol were added to a 20 mL scintillation vial. The vial was capped and stirred at 70 °C for 24 h. When the reaction was complete, the ethanol solvent was evaporated, yielding DMAHDM as a clear, colorless, and viscous liquid [33, 34]. DMAHDM was mixed with the acrylic resin liquid at DMAHDM/(MMA power + MMA liquid + DMAHDM) mass fractions of 1.5%. Higher DMAHDM mass fractions were not used because previous experiments showed a significant decrease in mechanical strength [30].

### Incorporation of Antibacterial MPC

The commercial protein-repellent monomer MPC (Sigma-Aldrich) was synthesized using the method reported by Ishihara et al. [35]. To fabricate the novel PMMA resin, the MPC powder was mixed with acrylic resin liquid at MPC/ (MMA powder + MMA liquid + MPC) mass fractions of 3%, according to our preliminary work [31]. Therefore, the following four groups were tested:

1. Nature Cryl™ MC powder and liquid control (termed “Commercial control”)
2. Nature Cryl™ MC powder and liquid + 3% MPC (“3% MPC”)
3. Nature Cryl™ MC powder and liquid + 1.5% DMAHDM (“1.5%DMAHDM”)
4. Nature Cryl™ MC powder and liquid + 3% MPC + 1.5% DMAHDM (“3%MPC + 1.5%DMAHDM”)

All the specimens were cured by heat polymerization at 65 ℃ for 20 min under 2.0 bar. Each of the aforementioned four groups was randomly divided for water-ageing at 1 d, 90 d, and 180 d, respectively. The water was changed once per week. Rectangular molds were used to fabricated uniform disks (2 × 2 × 25 mm) to investigate the mechanical properties (*n* = 10 for each group) [33]. For protein and biofilm tests (*n* = 6 for each group), each paste was placed into disk molds of 8 mm in diameter and 0.5 mm in thickness, and then incubated in distilled water and magnetically-stirred with a bar at a speed of 100 rpm for 1 h to remove any uncured monomers. The disks were then sterilized with ethylene oxide (Anprolene AN74i, Andersen, Haw River, NC) and de-gassed for 3 days for subsequent use [29].

### Mechanical testing

A computer-controlled Universal Testing Machine (AG-IS 500 N, Shimadzu Corporation, Kyoto, Japan) was used to measure the breaking strength of the specimens in three-point flexure with a span of 10 mm and a crosshead speed of 1 mm/min. Flexural strength (S) was calculated as: S = 3PmaxL/(2bh^2^), where Pmax is the maximum load on the load–displacement curve, L is the flexure span, b is the specimen width, and h is the specimen thickness [36]. The elastic modulus (E) was calculated by E = (P/d) (L^3^/[4bh^3^]), where load P divided by displacement and d is the slope in the linear elastic region of the load–displacement curve [37].

### Measurement of protein adsorption by the microbicinchoninic acid method

The micro bicinchoninic acid (BCA) method was used to determine the amount of protein adsorbed on the specimens [29, 37]. First, each sample was immersed in phosphate buffered saline (PBS) for 2 h. They were then placed into 4.5 g/L bovine serum albumin (BSA) (Sigma-Aldrich) solutions at 37 ℃ for 2 h. The disks then were washed for 5 min with fresh PBS by stirring at a speed of 300 rpm before immersing in sodium dodecyl sulfate (SDS) 1 wt.% in PBS. To detach the BSA adsorbed on the specimen surfaces, they were sonicated at room temperature for 20 min [29, 37]. To determine the BSA concentration in the SDS solution, a protein analysis kit (micro BCA protein assay kit, Fisher Scientific, Pittsburgh, PA, USA) was employed. From the standard curves, the amount of protein adsorbed on the disk was calculated.

### Saliva collection and biofilm inoculum

Human saliva was shown to be ideal for growing plaque microcosm biofilms in vitro, with the advantage of maintaining much of the complexity and heterogeneity of the dental plaque in vivo [38]. Saliva was obtained from ten healthy adult individuals. The donors should have natural dentitions, be free from active caries or periopathology, and stay away from antibiotics within the last 3 months, as stated in previous studies [19, 20]. They did not brush their teeth for 24 h and stopped any food and drink intake for 2 h before donating saliva. Stimulated saliva was collected and kept on ice. An equal volume of saliva from each of the ten donors was mixed together, diluted to 70% saliva and 30% glycerol, and stored at − 80 ℃ [35].

The growth medium was an artificial saliva named the McBain medium. The saliva-glycerol stock was added into the growth medium at 1:50 final dilution. The McBain medium contained mucin (type II, porcine, gastric) at a concentration of 2.5 g/L; tryptone, 2.0 g/L; yeast extract, 1.0 g/L; bacteriological peptone, 2.0 g/L; cysteine hydrochloride, 0.1 g/L; NaCl, 0.35 g/L, KCl, 0.2 g/L; CaCl_2_, 0.2 g/L; hemin, 0.001 g/L; vitamin K1, 0.0002 g/L, at pH 7 [21, 27]. Each resin disk was placed into a well of a 24-well plate. A saliva/McBain inoculum of 1.5 mL was added to each well and incubated in 5% CO_2_ at 37 ℃. After 8 h, the disks were transferred to new 24-well plates, filled with fresh medium and incubated for 16 h. The disks were then moved to new 24-well plates with new culture medium and cultured for 24 h. This totaled 2 d of incubation, which allowed microcosm biofilms to form, as shown previously [39].

### Live/dead biofilm staining assay

For the live/dead bacterial staining assay, specimens with 2-day biofilms were rinsed three times with PBS and stained using a BacLight live/dead kit (Molecular Probes, Eugene, OR, USA). When live bacteria were stained with Syto 9, green fluorescence was produced. Red fluorescence was produced when bacteria with compromised membranes were stained with propidium iodide. The stained disks were examined using an inverted epifluorescence microscope (Eclipse TE2000-S, Nikon, Melville, NY, USA). Three randomly chosen fields of view were photographed for each disk, yielding a total of 18 images for each group [35].

### MTT(3-[4,5-dimethylthiazol-2-yl]-2,5-diphenyltetrazoliumbromide) assay of metabolic activity

Specimens with 2-day biofilms were transferred to a new 24-well plate for an MTT assay to determine the metabolic property of the oral plaque biofilms. 1 mL of MTT dye (0.5 mg/mL MTT in PBS) was added to each well and incubated at 37 ℃ in 5% CO_2_ for 1 h. During this process, metabolically active bacteria reduced the MTT to purple formazan. After 1 h, the disks were transferred to a new 24-well plate, and 1 mL of dimethyl sulfoxide (DMSO) was added to each well. The plate was incubated for 20 min at room temperature in the dark. Then, 200 mL of the DMSO solution from each well was collected, and the absorbance at 540 nm was measured using a microplate reader (Spectra-Max M5, Molecular Devices, LLC., San Jose, CA, USA). A higher absorbance is related to a higher formazan concentration, which indicates a higher metabolic activity in the biofilm on the disk [29, 34].

### Colony-Forming Unit (CFU) counts of biofilms

To measure the biofilm CFU, disks with two-day biofilms were transferred into tubes with 2 mL of cysteine peptone water (CPW), and the biofilms were harvested by sonication for 5 min, followed by vortexing (Fisher) for 30 s. Three types of agar plates were prepared. Tryptic soy blood agar culture plates were used to determine total microorganisms. Mitis salivarius agar (MSA) culture plates with 15% sucrose were used to determine total streptococci. Selective agents crystal violet, potassium tellurite and trypan blue were added into MSA culture plates, which inhibit most gram-negative bacilli and most gram-positive bacteria except streptococci, thus enabling streptococci to grow. The third type of agar plate was MSA agar plus 0.2 units/mL of bacitracin, to determine mutans streptococci. This is because cariogenic mutans streptococci are resistant to bacitracin, hence this property can be used to isolate mutans streptococci from the highly heterogeneous oral microflora. The bacterial suspensions were serially diluted and spread onto agar plates for CFU analysis [26, 34].

### Statistical analysis

One-way and two-way analyses of variance (ANOVA) were performed to detect the significant effects of variables. Tukey’s multiple comparison test was used to compare the data at a *p* value of 0.05. Standard deviations (SD) served as an estimate for standard uncertainties associated with the measurements.

## Results

**Fig. 1:**
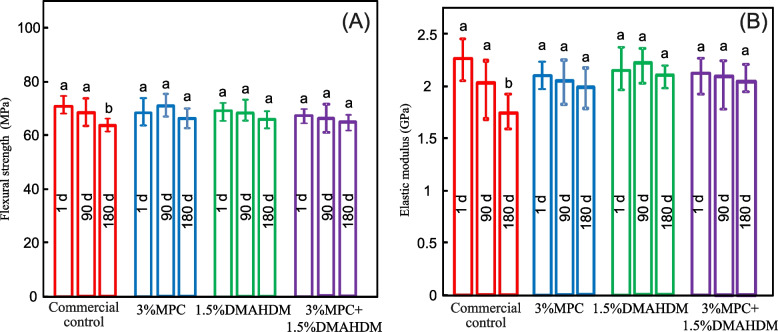
Mechanical properties of samples: **A** Flexural strength; and **B** elastic modulus (mean ± SD; *n* = 10). Bars with dissimilar letters indicate values that are significantly different from each other (*p* < 0.05)

Figure 1 shows the results of flexural strength and elastic modulus tests of the different groups (mean ± SD; *n* = 10). The strengths were not significantly different from each other (*p* > 0.1), except the control group after 180 d of water-ageing, which was significantly lower than that of the other group (*p* < 0.05). There was a 10% strength loss for the commercial PMMA resin during 180 days of water-ageing.

**Fig. 2:**
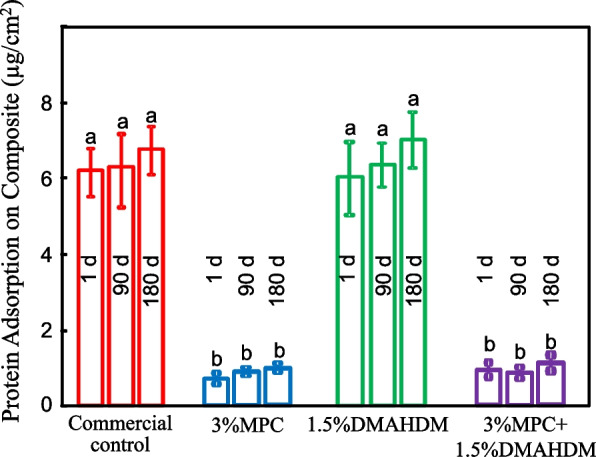
Protein adsorption on specimen surfaces (mean ± SD; *n* = 6). Bars with dissimilar letters indicate values that are significantly different from each other (*p* < 0.05)

The amount of protein adsorption on the acrylic resin disks were plotted in Fig. 2 (mean ± SD; *n* = 6). The amounts of protein adsorbed by the 3% MPC group decreased by approximately 80% compared with that of the control. Incorporating MPC into the acrylic resin greatly decreased the amount of protein adsorption, and there was no difference between 1 and 180 days. The 3% MPC group had the same protein adsorption as the 3% MPC + 1.5% DMAHDM group (*p* > 0.1), which indicated that MPC could repel protein, but DMAHDM had no effect on protein adsorption (*p* < 0.05).

**Fig. 3:**
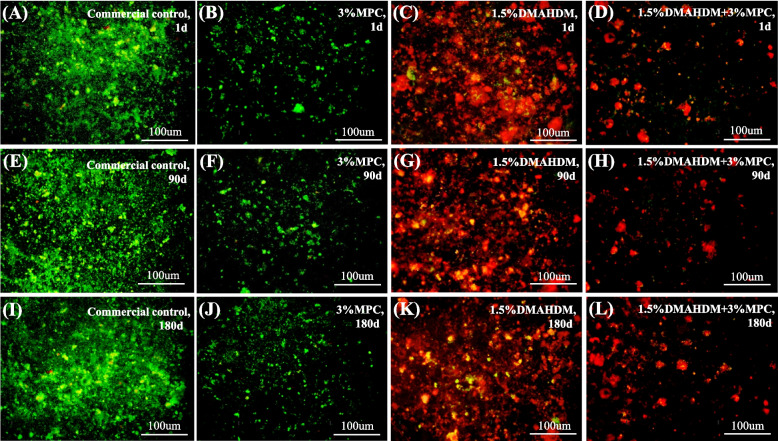
Representative live/dead images of two-day biofilms on specimens. Live bacteria are stained green, and dead bacteria are stained red

Figure 3 shows the live/dead staining images of the biofilms. The acrylic resin control was fully covered by primarily live bacteria, while the DMAHDM + MPC group had the least bacterial attachment and comprised mainly dead bacteria. The specimens of the 3% MPC group had much less bacterial adhesion, but the bacteria were mostly alive (green staining). In contract, the 1.5% DMAHDM group had substantial amounts of dead bacteria with red staining. There was no noticeable difference between 1 and 180 days for all the groups.

**Fig. 4:**
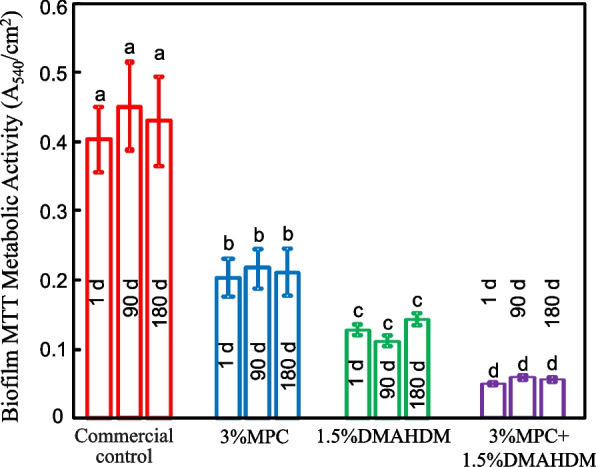
Metabolic activity of samples (mean ± SD; *n* = 6). Bars with dissimilar letters indicate values that are significantly different from each other (*p* < 0.05)

The quantitative metabolic activity determined using the MTT assay was plotted in Fig. 4 (mean ± SD; *n* = 6). Compared the control, adding DMAHDM into the acrylic resin significantly reduced the MTT absorbance (*p* < 0.05). Dual agents DMAHDM + MPC in the acrylic resin further substantially reduced the MTT absorbance (*p* < 0.05). The results demonstrated that combining DMAHDM and MPC in the same specimen exhibited the least metabolic activity by comparison with adding DMAHDM alone. Water-ageing for 180 days did not reduce the antibacterial efficacy compared with that at 1 d (*p* > 0.1).

**Fig. 5:**
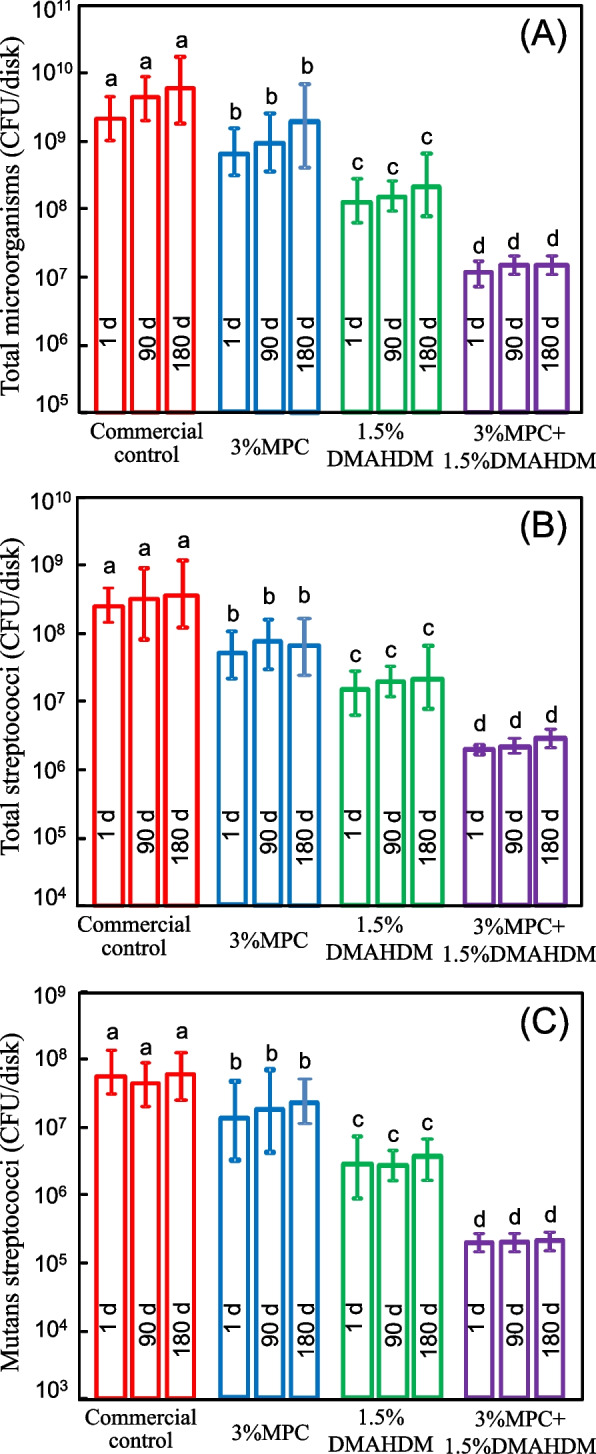
Colony-forming unit (CFU) counts for: **A** Total microorganisms; **B** total streptococci; and **C** mutans streptococci (mean ± SD; *n* = 6). Bars with dissimilar letters indicate values that are significantly different from each other (*p* < 0.05)

The biofilm CFU counts are shown in Fig. 5 for (A) total microorganisms, (B) total streptococci, and (C) mutans streptococci (mean ± SD; *n* = 6). DMAHDM, MPC, and DMAHDM + MPC greatly decreased the biofilm CFU both at 1 day and day 180 of water-aging compared with that of the control (*p* < 0.05). The incorporation of dual agents (DMAHDM + MPC) in the PMMA resin had a significantly stronger antibacterial effect than using DMAHDM alone, reducing the biofilm CFU by about two orders of magnitude compared with that of the commercial control (*p* < 0.05).

## Discussion

In dentistry, bacteria tend to accumulate and propagate on teeth or solid surfaces, which could cause biofilm-related side effects. A possible alternative to overcome this problem is the development of dental acrylic resins capable of inhibiting biofilm formation by adding antibiofilm agents. In general, antibacterial agents can be divided into soluble antibacterial agents and immobilizable antibacterial agents. The antibacterial effect of the leachable antibacterial agent is relatively short, which has certain impact on the mechanical property, and may even produce toxicity. While the insoluble antibacterial agents could be fixed by polymerization, which could lead to a long term antibiofilm effect. The polymerizable quaternary ammonium compounds are the most commonly used immobilizable antibacterial agents by reason of broad-spectrum antibacterial activity and good biocompatibility [19–23]. In the present study, a bioactive PMMA resin containing DMAHDM and MPC was successfully fabricated, which demonstrated excellent mechanical strength and durability, and maintained a potent anti-biofilm activity for 180 days of water-ageing. Our results proved the hypothesis that the PMMA–MPC–DMAHDM-containing resin imparted a strong antibacterial effect by greatly reducing biofilm viability and metabolic activity. The PMMA–MPC–DMAHDM-containing resin exhibited long-term antibacterial performance, with no significant difference between 1 d, 3 months, and 6 months. The mechanism of the antibacterial effect of QAMs is thought to be contact killing [25]. QAMs contain four organic groups linked to nitrogen, which are positively charged and can attract the negatively charged bacterial cell membrane. The electric balance of the cell membrane could then be disrupted, resulting in cytoplasmic leakage. Alternatively, QAMs with long lipophilic alkyl chains could bind to the cell wall components, resulting in the physical disruption of the membrane and bacterial cell death. Notably, the length of the hydrophobic chains can affect the antibacterial activity of QAMs. In theory, increasing the alkyl chain length would strengthen the bacteria-eradicating ability because of the stronger penetration property and better exposure of the quaternary ammonium sites. However, the chain length has a threshold value, beyond which the antimicrobial activity declines [40]. The optimal chain length is 16, which is long enough to penetrate the bacterial membrane without bending or curling. Thus, DMAHDM has the strongest antibacterial activity among the QAMs tested [40]. In the present study, the acrylic resin containing DMAHDM exhibited an excellent antibacterial performance by substantially reducing biofilm viability and the CFU count. The incorporation of DMAHDM reduced the biofilm activity of the commercial control by about 70% and decreased the biofilm CFU by about one order of magnitude (*p* < 0.05), both before and after water-aging for 180 days. The results suggested that DMAHDM could exhibit long-lasting bactericidal effects. The reason for adding DMAHDM to the resin to yield a long-term antibacterial property is that the antibacterial component in the polymer network is immobilized by polymerization of DMAHDM and does not leach out from cured acrylic resins [26, 41].

In addition to killing bacteria directly, reducing the formation of dental plaque is also an effective way to reduce the occurrence of caries. Protein adsorption to the pellicle is a prerequisite for the formation of plaque [42]. If the PMMA resin itself has protein repellent property, it could effectively reduce surface protein adhesion and bacterial accumulation, thereby inhibiting the formation of plaque at source and reducing the incidence of oral diseases. MPC, with a phospholipid polar group in the side chain, is established as a polymer with excellent biocompatibility and hydrophilic properties [43–46]. Most proteins prefer to bind to hydrophobic surfaces; therefore, the PMMA resin with hydrophilic MPC could reduce protein adhesion and bacterial attachment. In the present study, the amount of protein absorbed by the 3% MPC group was decreased by approximately 80% compared with that of the control, suggesting the excellent protein-repellent capability of the MPC-containing specimens. In addition, there was no significant difference between this effect on day 1 and day 180. MPC could copolymerize with the acrylic resin and become immobilized; therefore, it was present even after brushing and wear to continue detaching proteins.

Studies reported a synergistic effect of MPC and QAMs on antibiofilm properties [26, 43]. Thongthai et al. developed a novel surface coating composed of MDPB/MPC/BMA on PMMA resin, which exhibited both protein-repellent ability and bactericidal effects against *S. mutans* and effectively inhibited the formation of *S. mutans* biofilms [43]. The authors found that the number of viable *S. mutans* cells after incubation for 24 h in the MDPB/MPC/BMA group was lower than that in the MDPB/ BMA group, suggesting that the antibacterial efficacy was enhanced when both agents (protein-repellant MPC + antibacterial DMAHDM) were used in the same PMMA resin [43]. The results were similar to those of the present study. The biofilm CFU counts of the PMMA resin containing a single agent (3% MPC) decreased by about 50% compared with the control, and incorporating 1.5% DMAHDM alone in the resin reduced the biofilm CFU count of the commercial control by about one order of magnitude. However, when MPC and DMAHDM were both added into the PMMA resin, the biofilm CFU was reduced by about two orders of magnitude compared with that of control group. These results showed that the antibiofilm effect of DMAHDM was strengthened when combined with the protein-repellent MPC. The antibacterial effect of DMAHDM depends on direct contact with the biofilm; therefore, the contact-killing efficacy could be attenuated by salivary proteins attached on its surface. MPC can effectively resist protein adsorption on the acrylic resins; therefore, more direct resin-bacteria contact could occur, thus facilitating and enhancing the antibiofilm effect [26, 29, 43].

In the oral cavity, acrylic-based resins are exposed to a complex environment, which will result in deterioration of the material [47, 48]. Water is one of the main factors causing biodegradation. Water molecules can penetrate into the polymer network, occupy the spaces between the polymer chains, and break the adjacent chains, leading to a reduction in surface hardness and wear resistance [47, 49]. Moreover, the PMMA resin will expand after absorbing water, and thereby tiny cracks will occur inside the resin. When subjected to greater chewing pressure or external force, the resin will break from the site of these microcracks [49]. The deterioration of acrylic-based materials is not only related to physiochemical factors, such as water, temperature, and load, but also to biological factors, such as salivary proteins and bacterial metabolic activities. Bacteria can invade the acrylic resins through micropores, reducing the mechanical properties of the resins through enzyme activity or the production of volatile metabolites [48]. In this study, a three-point flexure test was used to determine the flexural strength and elastic modulus of the specimens with or without DMAHDM and/or MPC. In order to simulate the oral environment, thermal-cycling and water immersing were conducted. A two-temperature thermal cycler was used, and the two water baths were maintained at temperatures of 5 °C and 55 °C, respectively. During the immersion, specimens were placed in a constant temperature water at 37 º C. Strength loss for the antibacterial acrylic resins incorporating DMAHDM, MPC, and DMAHDM-MPC was not statistically significant, both before and after water-aging for 180 days. However, the strength of control group after 180 d of water immersion was significantly lower than that of the other groups. These results suggested that the wear of the PMMA resin was significantly reduced by the use of DMAHDM and/or MPC. It is reported that MPC can lubricate the surface of an acrylic resin base, thereby leading to the resistance to mechanical stress [26, 50]. Furthermore, the reduced protein adhesion and bacterial attachment could enhance mechanical resistance to a certain extent [26]. The appliance with antibacterial and anti-protein repellent effect can significantly reduce the formation of dental plaque, thereby reducing the occurrence of oral diseases such as caries.

The new bioactive PMMA resin containing double agents of MPC and DMAHDM offers a possibility of reduction in the incidence of oral diseases by greatly reducing protein adsorption and biofilm growth. In addition, as PMMA is also a very popular biomaterial used for the fixation of artificial joints, the addition of double agents MPC and DMAHDM to PMMA could reduce the risk of infection in implant surgery [51]. The present study focused on the effects of the 180 days of water-ageing treatment on the strength and antibacterial properties of PMMA resin in vitro, without considering the effect of toothbrushing on the biofilm. Further study is needed to investigate the durability of the MPC-DMAHDM composite in reducing biofilm growth and protein adsorption under repeated biofilm challenges.

## Conclusions

The present study investigated the durability of PMMA resin with a combination of protein-repellent and antibacterial capabilities during water-aging. While a commercial acrylic resin lost 10% of its mechanical strength after 180 days of water-ageing, the new antibacterial resin containing DMAHDM and/or MPC exhibited long-lasting strength after water immersion. The synergistic effect of MPC and DMAHDM on antibiofilm properties meant that the PMMA resin achieved the strongest antibacterial effects after adding both MPC and DMAHDM together. The novel protein-repellent and antibacterial PMMA resin has potential for acrylic-based resin wearers to inhibit caries in the long erm.

## Data Availability

The data of this study are available from corresponding author on reasonable request.

## Abbreviations

PMMA: Polymethyl methacrylate
MPC: 2-Methacryloyloxyethyl phosphorylcholine (MPC)
DMAHDM: Quaternary ammonium dimethylaminohexadecyl methacrylate
CFU: Colony Forming Unit
